# 975. Age and Body Mass Index Impact Optimal Vancomycin Model Selection for Bayesian Dosing: Results from a Large Multi-site Retrospective Study of Adult Patients

**DOI:** 10.1093/ofid/ofad500.030

**Published:** 2023-11-27

**Authors:** Maria-Stephanie Hughes, Tiffany Lee

**Affiliations:** InsightRX, Boston, MA; InsightRX, Boston, MA

## Abstract

**Background:**

Vancomycin population pharmacokinetic (popPK) models embedded in Bayesian software are crucial to informing optimal initial dosing. Since pharmacokinetics (PK) vary based on patients’ age and body mass index (BMI), pharmacists are faced to select the best model amongst many for their patients. To aid in model selection, we used a large multi-site database to determine which models were the most accurate across age and BMI categories.

**Methods:**

Data on adult patients receiving intravenous vancomycin therapy during January 1, 2022 - December 31, 2022, at healthcare organizations across the U.S. were queried from a Bayesian software database. Data collected included age, weight, height, serum creatinine, sex, vancomycin doses received, and vancomycin concentration levels. The *a priori* root mean square error (RMSE) for six well-performing and previously validated models was calculated for each patient to measure what the accuracy would have theoretically been if each were used. The average RMSE value per model was calculated and compared between age and BMI categories shown in Table 1. Each age-BMI combination’s most accurate model was reported.

**Figure:**
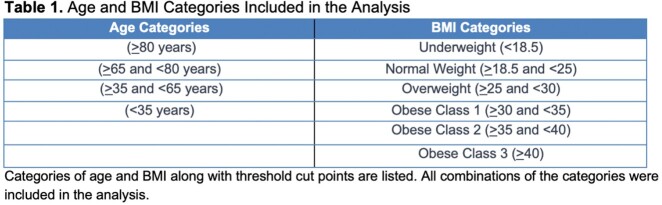

**Results:**

There were 79,600 patient treatment courses included across 84 health systems. The results for the most accurate model for each age-BMI combination are reported in Table 2. The lowest RMSE was 3.91 mg/L using the modified Goti model in patients > 80 years with BMI < 18.5 kg/m^2^. The highest RMSE was 7.86 mg/L using the Carreno model in patients < 35 years with BMI > 30 and < 35 kg/m^2^. A relationship was observed where older age categories lead to better model performance.

**Figure:**
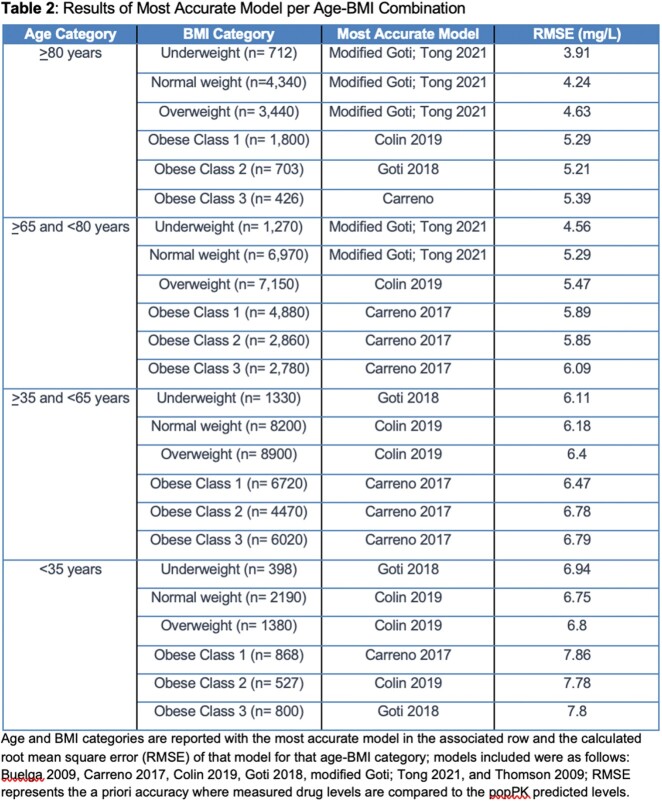

**Conclusion:**

Age and BMI categories influenced which vancomycin popPK models performed best *a priori* within a Bayesian software when studied within a very robust dataset. These findings may help pharmacists choose which model to use for the most accurate PK calculations and thus inform optimal initial pop-PK vancomycin dosing. It also highlights that adults under 35 years, which tend to be underrepresented in model development populations, showed higher prediction error. Further research is needed on how to best implement these findings into practice.

**Disclosures:**

**Maria-Stephanie Hughes, PharmD**, InsightRX: Stocks/Bonds **Tiffany Lee, PharmD**, InsightRX: Employment

